# Metabolite‐Driven Biological Activity of *Mandragora autumnalis* and *Spartium junceum*: A Comparative LC‐MS/MS Study on Antioxidant, Cholinesterase, and Carbonic Anhydrase Inhibition

**DOI:** 10.1002/open.70269

**Published:** 2026-08-13

**Authors:** Kubra Aslan, Esra Elbir, Yusuf Bicakci, Silanur Ozdogan, Mustafa Abdullah Yilmaz, Ilhami Gulcin

**Affiliations:** ^1^ Department of Chemistry Faculty of Science Ataturk University Erzurum Türkiye; ^2^ Department of Molecular Biology and Genetics Faculty of Science Ataturk University Erzurum Türkiye; ^3^ Department of Analytical Chemistry Faculty of Pharmacy Dicle University Diyarbakir Türkiye; ^4^ Rectorate of Agri Ibrahim Cecen University Agri Türkiye

**Keywords:** antioxidant, enzyme inhibition, *Mandragora autumnalis*, neurodegenerative disorders, *Spartium junceum*

## Abstract

In this study, the targeted phytochemical profiles and multi‐target biological activities of *Mandragora autumnalis* L. and *Spartium junceum* L. were evaluated using liquid chromatography–tandem mass spectrometry (LC‐MS/MS), antioxidant, enzyme inhibition, and antimicrobial assays. LC‐MS/MS analysis showed that *M. autumnalis* predominantly contained low‐molecular‐weight phenolic acids, particularly protocatechuic acid (0.6850 µg/mg), whereas *S. junceum* was richer in flavonoids and isoflavonoids, especially genistein (1.8280 µg/mg) and cynaroside (1.5240 µg/mg). *M. autumnalis* showed stronger antioxidant activity, with a DPPH IC_50_ value of 60.16 µg/mL and a FRAP absorbance of 2.098 ± 0.042 at 45 µg/mL, whereas *S. junceum* showed weak DPPH radical scavenging activity (IC_50_: 813.50 µg/mL). *M. autumnalis* also exhibited stronger acetylcholinesterase and carbonic anhydrase inhibition, on the other hand *S. junceum* displayed a butyrylcholinesterase‐oriented inhibitory profile. Preliminary antimicrobial screening revealed inhibition zones of 11–13 mm against selected bacterial strains. Overall, the findings suggest that low‐molecular‐weight phenolic acids may contribute to the biological activity of *M. autumnalis*, whereas flavonoids and isoflavonoids may support the characteristic inhibitory profile of *S. junceum*, highlighting the importance of qualitative metabolite composition in shaping extract bioactivity.

## Graphical abstract

Low‐molecular‐weight phenolic acids in *M. autumnalis* were associated with antioxidant activity, AChE, and hCA inhibition effects, whereas flavonoid‐rich *S. junceum* displayed BChE‐oriented inhibition effect and antibacterial activity. LC‐MS/MS‐based metabolite profiling linked distinct chemical compositions to bioactivity patterns, supporting further comparative bioactivity‐guided investigation of these extracts.

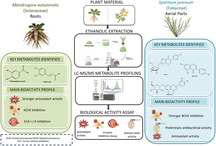

## 1 Introduction

Plants remain important sources of bioactive secondary metabolites because of their structural diversity and multi‐target biological potentials. Among these metabolites, phenolic acids, flavonoids, and related compounds are particularly relevant due to their antioxidant, antimicrobial, anti‐inflammatory, and enzyme‐inhibitory properties [1]. However, the biological activity of plant extracts depends not only on their total phenolic or flavonoid content, but also on their qualitative metabolite composition, chemical diversity, and possible synergistic interactions among constituents [1, 2]. Therefore, targeted phytochemical profiling combined with biological activity assays is essential for understanding the relationship between chemical composition and bioactivity.

*Mandragora autumnalis* L. (Solanaceae) and *Spartium junceum* L. (Fabaceae) are traditionally recognized as toxic species, mainly due to their alkaloid contents [1, 2]. Despite their toxicity, both plants have also been associated with traditional medicinal uses. *M. autumnalis* has historically been used for sedative, analgesic, antispasmodic, and hypnotic purposes, whereas *S. junceum* has been reported for diuretic, digestive, anti‐inflammatory, antiulcer, and analgesic applications [1, 2, 3, 4]. This combination of ethnomedicinal relevance, toxicity, and phytochemical diversity makes them suitable candidates for comparative bioactivity screening. Although both species are known for their toxic alkaloid contents, the present study specifically focuses on their phenolic acids, flavonoids, and related secondary metabolites, as these compounds were the target of the LC‐MS/MS profiling strategy and are directly relevant to the evaluated antioxidant, antimicrobial, and enzyme inhibitory effects [3, 4, 5, 6].

Although *M. autumnalis* and *S. junceum* have been individually investigated for selected biological effects, comparative multi‐target studies integrating LC‐MS/MS‐based metabolite profiling with antioxidant, cholinesterase, carbonic anhydrase inhibition, and antimicrobial assays remain limited.

In this study, phytochemical characterization was performed using a targeted LC‐MS/MS profiling strategy focused on the detection and quantification of selected phenolic acids, flavonoids, and related secondary metabolites using reference standards and multiple reaction monitoring transitions. This approach allowed a comparative evaluation of the metabolite composition of *M. autumnalis* and *S. junceum* and provided a chemical basis for interpreting their biological activity profiles. To the best of our knowledge, this is the first study to comparatively evaluate the LC‐MS/MS‐based phytochemical profiles of *M. autumnalis* and *S. junceum* together with their acetylcholinesterase (AChE), butyrylcholinesterase (BchE), human carbonic anhydrases I (hCA I), and II (hCA II) inhibitory effects [6].

## 2 Materials and Methods

### 2.1 Materials and Reagents

In this study, both plant species collected from different locations in the Aegean region of Türkiye were used as plant materials. The aerial parts of *S. junceum* L. were collected from Buca, İzmir, Türkiye, in April 2025 (38°22′53.1″N, 27°09′46.5″E), whereas the roots of *M. autumnalis* were collected from Denizli, Türkiye, in April 2025 (37°47′00.0″N, 29°05′41.0″E). The plant specimens were taxonomically identified and scientific names of the species were further verified using the GBIF and Catalogue of Life databases. However, no herbarium voucher specimens were deposited, and therefore no voucher specimen number is currently available.

The bacterial reference strains used in this study, namely *Staphylococcus aureus* ATCC 25923, *Klebsiella pneumoniae* ATCC 3883, *Pseudomonas aeruginosa* ATCC 27853, and *Escherichia coli* ATCC 8739, were obtained from the American Type Culture Collection (ATCC). Butylated hydroxytoluene (BHT), butylated hydroxyanisole (BHA), α‐tocopherol, 1,1‐diphenyl‐2‐picrylhydrazyl (DPPH), and the other chemicals used in the study were purchased from Sigma–Aldrich (Steinheim, Germany). Standard phenolic compounds used in LC/MS–MS calibration (quinic acid, gallic acid, fumaric acid, aconitic acid, protocatechuic acid, epigallocatechin, gentisic acid, catechin, chlorogenic acid, tannic acid, protocatechuic aldehyde, epigallocatechin gallate, epicatechin, 4‐OH‐benzoic acid, vanillic acid, syringic acid, caffeic acid, vanillin, daidzin, syringaldehyde, epicatechin gallate, ferulic acid, sinapic acid, *p*‐coumaric acid, coumarin, cynaroside, salicylic acid, rutin, miquelianin, isoquercitrin, *o*‐coumaric acid, hesperidin, genistin, ellagic acid, rosmarinic acid, cosmosiin, astragalin, quercitrin, nicotiflorin, daidzein, fisetin, quercetin, luteolin, naringenin, hesperetin, apigenin, genistein, kaempferol, chrysin, amentoflavone, and acacetin) were purchased from Sigma–Aldrich (Steinheim, Germany). 1,5‐Dicaffeoylquinic acid and the internal standards of rutin‐D3, ferulic acid‐D3, and quercetin‐D3 were obtained from TRC (Toronto, Ontario, Canada). Acetylcholinesterase (AChE) enzyme obtained from *Electrophorus electricus* (electric eel), and butyrylcholinesterase (BChE) from equine serum were used in lyophilized powder form, type VI‐S (200–1000 U/mg protein) [7].

### 2.2 Preparation of Plant Extracts

**FIGURE 1:**
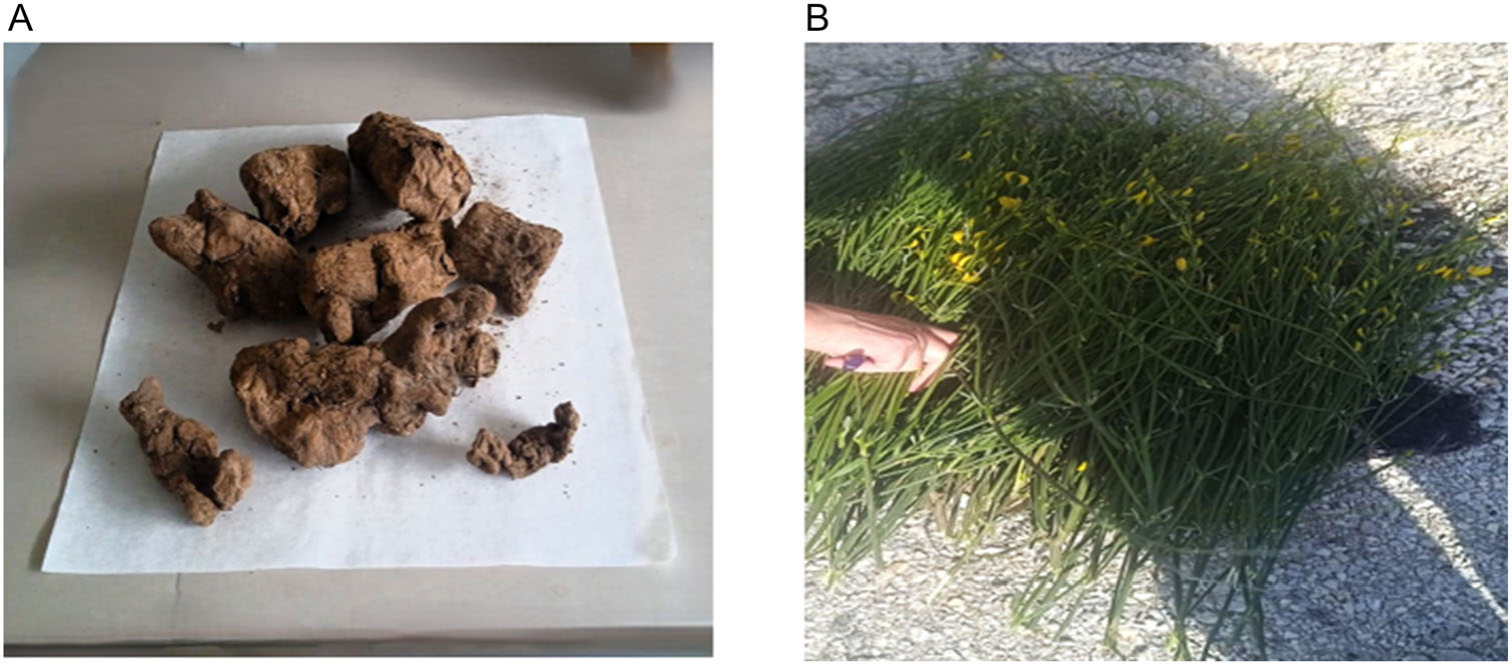
The plant labeled (A) represents the root of *M. autumnalis*, while the plant labeled (B) represents the aerial parts of *S. junceum*.

The two different plant materials obtained were first dried in the shade at room temperature (Figure 1A,B). After the drying process was completed, the *S. junceum* and *M. autumnalis* samples were ground to a particle size of 0.5–10 mm using a grinder. From the ground plant samples, 10 g were weighed, 100 mL of ethanol was added, and the mixture was extracted for 5 h on a magnetic stirrer. At the end of the process, ethanol was removed using a rotary evaporator (Heidolph Hei‐Vap PrecisionG3 Rotary Evaporator, Schwabach, Germany). From the obtained dry extracts, 80 mg were separated for LC‐MS/MS analyses. The remaining ethanol and dimethyl sulfoxide (DMSO) extract solutions were prepared to be used in antioxidant capacity, enzyme inhibition, and antimicrobial susceptibility tests.

### 2.3 Phytochemical Profiling

#### 2.3.1 Total Flavonoid Content (TFC)

This analysis was carried out by modifying previous methods in the literature [5]. The TFC was determined using the Al(NO_3_)_3_ colorimetric method. Three different concentrations (15–45 μg/mL) of ethanol extracts were mixed with 1.5 mL of 95% methanol. Then, 0.5 mL of 10 M potassium acetate, 2.3 mL of distilled water, and 1.5 mL of 10% aluminum nitrate were added to the reaction mixture, and the tubes were vortexed. The prepared mixtures were incubated at room temperature for 25 min. After the incubation period, the absorbance of the samples was measured at 415 nm using a Shimadzu UV‐1800 UV Spectrophotometer. The results were expressed as mg quercetin equivalents per gram of extract (mg QE/g extract) [6].

#### 2.3.2 Total Phenolic Content (TPC)

The TPC was determined using the Folin–Ciocalteu reagent by the colorimetric method [7]. For the analysis, the extracts were prepared at three different concentrations (15–45 μg/mL). Diluted extract samples in 0.5 mL of total volume were mixed with Folin–Ciocalteu reagent (FCR, 1.0 mL), and the mixture was allowed to react for 5 min. At the end of the period, 0.5 mL sodium carbonate (1%) solution was added to the mixture, and the final volume was adjusted to 2.0 mL with ethanol. After 2 h of incubation in the dark at room temperature, measurements were performed at a wavelength of 750 nm using a Shimadzu UV‐1800 UV Spectrophotometer. The results were calculated from the gallic acid calibration curve and expressed as mg gallic acid equivalents per gram of dry extract (mg GAE/g extract) based on the standard calibration curve prepared within the concentration range of 0–200 μg/mL [6, 8].

#### 2.3.3 Determination of Secondary Metabolite Content by LC‐MS/MS

This study was carried out in accordance with the most recent methods applied in the current literature. LC‐MS/MS analyses were performed by the Central Research Laboratory of Dicle University. Phytochemical profiling of the extracts was carried out using an ultrahigh‐performance liquid chromatography system coupled with tandem mass spectrometry (UHPLC‐MS/MS) by monitoring 53 target analytes. The analytical method was based on a previously validated procedure and adapted for the analysis of the ethanol and aqueous extracts of the studied samples. Quantitative measurements were performed on a Shimadzu Nexera UHPLC system interfaced with an LCMS‐8040 triple quadrupole mass spectrometer. The chromatographic unit was equipped with a binary pump, autosampler, column oven, and online degassing module. The method validation parameters were detailed in Table S1 [9, 10].

The separation conditions were optimized to achieve high sensitivity and to minimize matrix‐related signal suppression. An Agilent Poroshell 120 EC‐C18 column (150 mm × 2.1 mm, 2.7 μm) was used at 40 °C. Gradient elution was applied using water containing 5 mM ammonium formate and 0.1% formic acid as mobile phase A and methanol containing 5 mM ammonium formate and 0.1% formic acid as mobile phase B. The flow rate was maintained at 0.5 mL min^−1^, and the injection volume was 5 μL [9].

Mass spectrometric detection was performed with an electrospray ionization source operating in both positive and negative ionization modes. Quantification of the analytes was achieved in MRM mode by selecting the most sensitive precursor‐to‐product ion transitions. The optimized MS parameters included a drying gas flow of 15 L min^−1^, nebulizing gas flow of 3 L min^−1^, a desolvation line temperature of 250 °C, a heat block temperature of 400 °C, and an interface temperature of 350 °C. All data acquisition and processing steps were carried out using LabSolutions software [9].

#### 2.3.4 Determination of Antioxidant Activity

Antioxidant activity was determined through two different principles as radical scavenging testing and reducing ability testing. Certain concentrations of the extracts samples were prepared and subjected to the corresponding tests with parallel testing of standard antioxidants including ascorbic acid, BHA, BHT, α‐tocopherol, and Trolox. Although all reference antioxidants were considered during method application, the final tables report only those standards that provided reliable and reproducible responses for the corresponding assay. Therefore, the reference compounds differ among assays depending on their analytical suitability under the specific reaction. The use of three concentrations (15, 30, and 45 µg/mL) was based on a previously optimized screening protocol routinely applied in our laboratory for plant‐derived extracts. This concentration interval was selected because it falls within the linear and reproducible response range of the applied spectrophotometric assays and allows reliable comparison between samples and reference antioxidants. In previous applications of this protocol, higher extract concentrations were more likely to cause matrix‐related interference, including intrinsic extract color, turbidity, and absorbance saturation. Therefore, the selected range was considered appropriate for comparative antioxidant screening rather than exhaustive dose‐response modeling. In the radical scavenging assay, the results were expressed as the IC_50_ value in μg/mL, and the IC_50_ values were calculated from the following formula:

$$IC_{50}=(0.5-a)/b$$

In the reducing power‐based assays, the results were expressed as absorbance ± SD because the primary aim was to compare the reducing capacities of the extracts with reference antioxidants under identical experimental conditions. This absorbance‐based expression has been widely used in reducing power assays, where higher absorbance values directly indicate stronger electron‐donating capacity [10, 11, 12, 13, 14, 15, 16]. Standard equivalent‐based reporting may facilitate inter‐study comparison; however, the absorbance‐based format was preferred here to allow direct comparison among the extracts and standards tested in parallel under the same assay conditions.

#### 2.3.5 Radical Scavenging Assays

##### 2.3.5.1 1,*1‐Diphenyl‐2‐Picrylhydrazyl* (*DPPH*) *Assay*

Radical scavenging activity was determined using the widely applied 1,1‐diphenyl‐2‐picrylhydrazyl (DPPH) method [17]. Prior to analysis, a 0.1 mM DPPH radicals solution was freshly prepared in ethanol and kept in the dark overnight to allow complete dissolution and stabilization of the absorbance before use. The plant extracts were prepared at three different concentrations, ranging from 15 to 45 μg/mL. The resulting mixture was incubated at 30 °C for 30 min. After the incubation period, the absorbance of the samples was measured at 517 nm using a Shimadzu UV‐1800 UV Spectrophotometer [18].

#### 2.3.6 Reducing Capacity Assays

##### 2.3.6.1 *Cupric Ions* (*Cu* ^*2+*^) *Reducing Antioxidant Power* (*CUPRAC Assay*)

Based on the technique of Apak et al., 1.0 mM copper(II) chloride (CuCl_2_), neocuproine (7.5 mM), and ammonium acetate buffer (1.0 M, pH 6.5) were added to the reaction tubes at equal volumes [19]. Plant extracts at three different concentrations (15–45 μg/mL) were then added to this prepared mixture, and the final volume was adjusted to 2.0 mL with distilled water. Under these conditions, the final concentrations of CuCl_2_, neocuproine, and ammonium acetate buffer in the reaction mixture were 0.25, 1.875 mM, and 0.25 M, respectively. The mixture was incubated at room temperature for 30 min. At the end of the incubation period, the absorbance of the samples was measured at 450 nm. The obtained data were evaluated by comparison with the activities of the standard antioxidants (Ascorbic acid, BHT, BHA, Trolox, and α‐Tocopherol).

##### 2.3.6.2 Ferric Reducing Antioxidant Power (FRAP Assay).

The FRAP reagent was prepared by mixing 10 mM TPTZ (in 0.4 mM hydrochloric acid), 20 mM iron(III) chloride (FeCl_3_), and 0.3 M sodium acetate buffer (pH 3.6) at a ratio of 1:1:10, respectively. Plant samples were prepared in the concentration range of 15–45 μg/mL [6]. The prepared tubes were incubated at 37 °C for 30 min. After incubation, the absorbance was measured at 593 nm. The obtained values were compared with the absorbance values of standard antioxidants (Ascorbic acid, BHT, BHA, Trolox, and α‐tocopherol) to determine the reducing capacity of the sample.

##### 2.3.6.3 Fe^3+^‐Reducing Assay

Extract samples were prepared at three different concentrations (15–45 μg/mL), and 0.75 mL of each extract was mixed with 1.25 mL of 0.20 M PBS (pH 6.6) and 1% (w/v) potassium ferricyanide in distilled water. The mixture was then incubated at 50 °C for 30 min in the dark after adding 10% (w/v) trichloroacetic acid. The incubation at 50 °C was applied according to the conventional Fe^3+^ reducing power assay protocol to facilitate the reduction of the ferricyanide complex by electron‐donating antioxidants and to ensure sufficient color development before absorbance measurement [20]. Subsequently, 0.25 mL of 0.1% iron(III) chloride solution was added to the reaction. The absorbance was measured at 700 nm. The obtained results were compared with the absorbance values of the standard antioxidants BHT, BHA, and Trolox [6].

#### 2.3.7 Enzyme Inhibition Studies

##### 2.3.7.1 AChE and BChE Inhibition

AChE and BChE enzyme inhibition activities were determined by modifying the Ellman spectrophotometric method [19]. Acetylthiocholine iodide and butyrylthiocholine iodide were used as substrates for AChE and BChE, respectively. In the inhibition assays, different concentrations of the ethanolic extracts of *M. autumnalis* and *S. junceum* were added to the reaction mixture. A 10 mM DTNB stock solution and a 10 mM substrate stock solution were used, and 50 µL of each solution was added to the reaction mixture, giving final concentrations of 0.5 mM DTNB and 0.5 mM substrate in a final reaction volume of 1 mL. The reaction was initiated by the addition of the enzyme solution, and the change in absorbance was monitored at 412 nm for 3 min against the control mixture. The percentage inhibition was calculated by comparing enzyme activity in the presence of the extracts with that of the inhibitor‐free control. Donepezil was used as the reference inhibitor [21].

##### 2.3.7.2 hCA I and hCA II Isoenzyme Inhibition

Human carbonic anhydrase I and II (hCA I and hCA II) isoenzymes were purified in laboratory conditions using Sepharose 4B‐L‐tyrosine‐sulfanilamide affinity chromatography, and the purity of the enzyme was characterized by the SDS‐PAGE method [10, 22, 23]. The esterase activity of the CA enzyme is based on the formation of the yellow‐colored *p*‐nitrophenol product at pH 7.5 as a result of substrate hydrolysis. In the inhibition assays, different concentrations of *M. autumnalis* and *S. junceum* extracts and the enzyme solution were added to the reaction mixture. (Various concentrations of the samples in 0.05 M Tris‐SO_4_ at pH 7.4, 0.07 mM of *p*‐nitrophenylacetate (in 1:25 acetone/water). Following the initiation of the reaction, the esterase activity was measured at 348 nm for 3 min. Acetazolamide (AZA) was used as the reference inhibitor [21].

#### 2.3.8 Preliminary Antimicrobial Screening Assay

Ethanol extracts of *M. autumnalis* and *S. junceum* were tested using the disk diffusion method as previously applied experiments [6]. The bacterial strains selected for the study included *S. aureus* ATCC 25923, *K. pneumoniae* ATCC 3883, and *P. aeruginosa* ATCC 27853. Before inoculation onto Nutrient agar surfaces, bacterial cells were transferred to 0.9% NaCl solution and diluted to 0.5 McFarland (approximately 1.5 × 10^8^ CFU/mL). Sterile 6 mm paper discs were loaded with extract solutions in 20 µL aliquots under aseptic conditions. The amounts of extract loaded onto each disc was adjusted to obtain the desired final doses expressed as µg/disc. When a higher dose was required, the discs were allowed to dry after the first loading step and then reloaded with an additional 20 µL aliquot until the target amount per disc was reached. Thus, the final extract amounts were expressed as µg/disc rather than µg/mL. The plates were inverted and incubated at 37 °C for 24 h [24]. Following incubation, the diameters of the inhibition zones around the discs were measured and recorded [25]. DMSO and tetracycline‐loaded discs were used as controls [6].

### 2.4 Statistical Analysis

All experiments were performed in triplicate, and the obtained results were presented as Mean ± SD. Statistical differences between the data were evaluated using Two‐Way ANOVA followed by Tukey's post hoc test, and *p* < 0.05 was accepted as statistically significant. Calculations and graphical analyses were conducted using GraphPad Prism 8.4.0 statistical software.

## 3 Results and Discussion

### 3.1 Phytochemical Analysis

Total phenolic and flavonoid contents are widely accepted as important indicators of the phytochemical richness of plant extracts and are closely associated with their antioxidant and enzyme inhibitory potentials [14]. They are also commonly used as preliminary screening tools prior to the application of more selective, sensitive, and expensive techniques such as LC‐MS/MS. In the present study, the ethanolic extracts of *M. autumnalis* and *S. junceum* were evaluated using the Folin–Ciocalteu and Al(NO_3_)_3_ colorimetric methods [26, 27, 28]. After extraction and solvent evaporation, the dry extract yields of *S. junceum* and *M. autumnalis* ethanol extracts were calculated as 5.87% and 2.66%, respectively, relative to the initial dry plant material. The TPC and TFC values of *M. autumnalis* were found to be 308.08 ± 17.08 mg GAE/g extract and 66.64 ± 1.22 mg QE/g extract, respectively, while those of *S. junceum* were 245.51 ± 8.52 mg GAE/g extract and 60.34 ± 0.65 mg QE/g extract. These results indicate that *M. autumnalis* possesses a higher phenolic and flavonoid accumulation capacity than *S. junceum*, which may explain its comparatively stronger biological activity [17, 29].

Albahri et al. (2025) reported considerably lower TPC and TFC values for the ethanolic leaf extract of *M. autumnalis* (58.98 ± 7.40 mg GAE/g and 36.47 ± 0.87 mg QE/g, respectively), suggesting that the phytochemical accumulation in this species is a dynamic process influenced not only by plant organ but also by environmental factors such as geographical origin and harvesting period [4]. This organ‐dependent metabolite distribution is consistent with the fact that the roots are mainly characterized by tropane alkaloids, whereas the aerial parts are richer in phenolic acids and flavonoids. However, the available data on the comparative phenolic and flavonoid profiles of different plant parts are still limited, and further detailed investigations are required to clarify their distribution patterns [3].

Similarly, in *S. junceum* extracts, the highest TPC and TFC values were reported in the ethanolic extract (38 ± 0.03 mg GAE/g and 24 ± 0.03 mg RE/g), whereas the lowest values were detected in the aqueous legumes extract (13 ± 0.03 mg GAE/g and 6 ± 0.05 mg RE/g), confirming the strong influence of plant part and extraction solvent on phenolic recovery. When compared with these literature data, the markedly higher phenolic and flavonoid contents obtained in the present study suggest that the studied samples are particularly rich in secondary metabolites, which may be attributed to differences in ecological conditions, plant material, or extraction efficiency. This high phenolic accumulation also supports the strong antioxidant and enzyme inhibitory effects observed for the investigated extracts.

**FIGURE 2:**
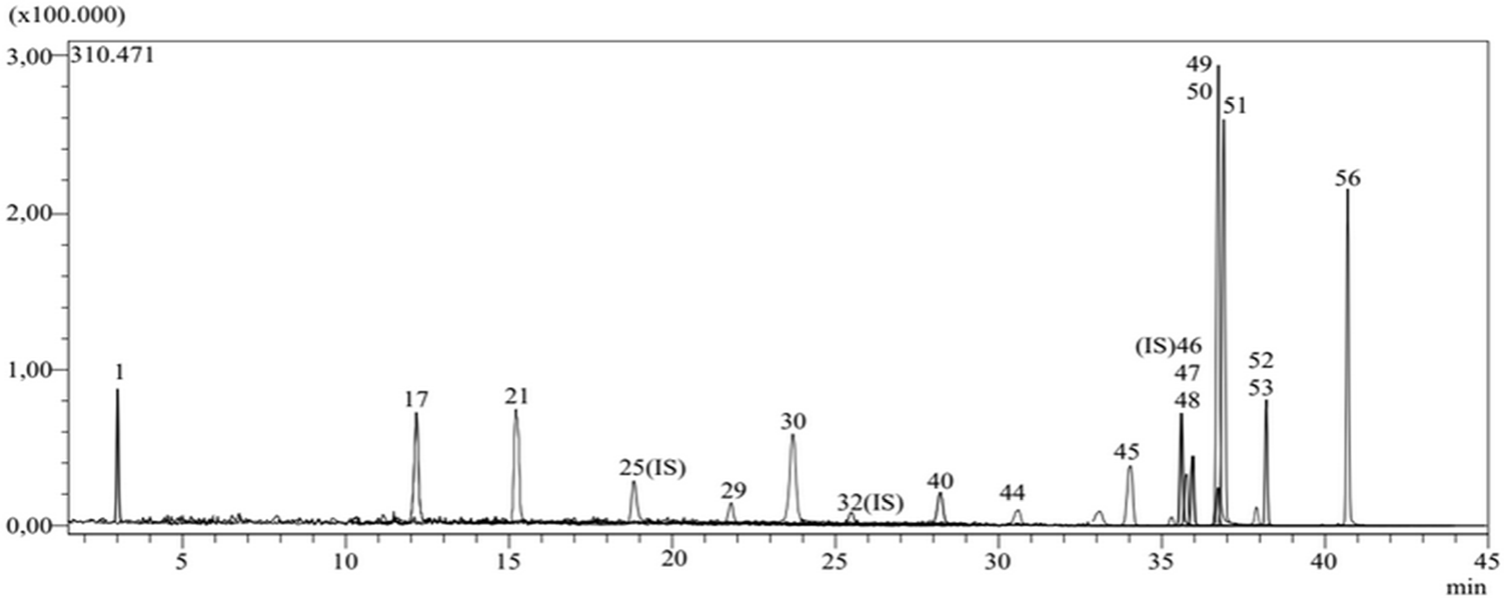
LC‐MS/MS chromatogram of the *S. junceum* ethanol extracts.

**FIGURE 3:**
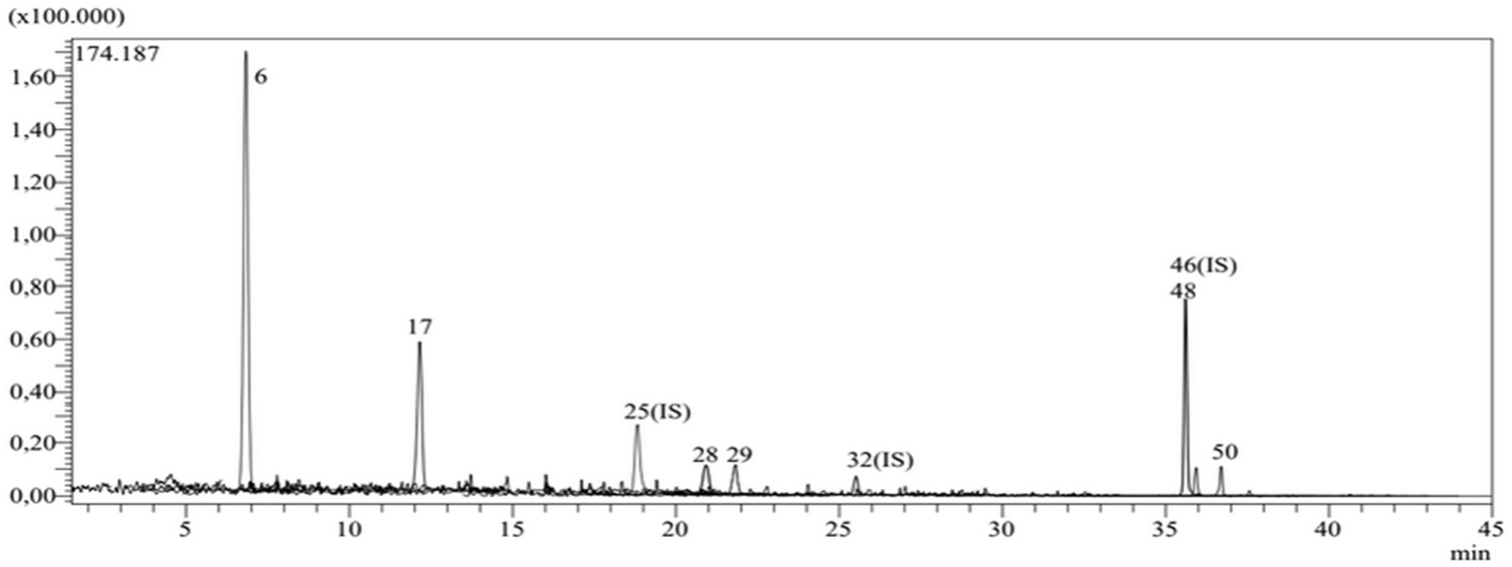
LC‐MS/MS chromatogram of the *M. autumnalis* ethanol extracts.

The detailed phytochemical composition of *S. junceum* and *M. autumnalis* was investigated through the LC‐MS/MS method by comparison with 53 phytochemical standards reported with their most significant biological activities [28]. Since important biological activities of natural compounds, such as antioxidant, antimicrobial, and anti‐inflammatory properties, are closely associated with phenolic content, the analysis of these compounds is essential for determining the value of herbal extracts for applications in the food, pharmaceutical, and cosmetic industries. The compounds identified in the aerial parts of *S. junceum* and their concentrations were as follows: quinic acid (0.4673 µg/mg), caffeic acid (0.1240 µg/mg), daidzin (1.0920 µg/mg), salicylic acid (0.0460 µg/mg), cyranoside (1.5240 µg/mg), cosmosiin (0.9550 µg/mg), fisetin (0.4830 µg/mg), daidzein (0.4830 µg/mg), quercetin (0.0220 µg/mg), naringenin (0.1660 µg/mg), hesperetin (0.0110 µg/mg), luteolin (0.8380 µg/mg), genistein (1.8280 µg/mg), kaempferol (0.0080 µg/mg), apigenin (0.2790 µg/mg), and acacetin (0.2330 µg/mg). Detailed quantitative results are presented in Table 1. In the roots of *M. autumnalis*, six compounds were detected, namely protocatechuic acid (0.6850 µg/mg), caffeic acid (0.1080 µg/mg), coumarin (0.0280 µg/mg), salicylic acid (0.0570 µg/mg), naringenin (0.0050 µg/mg), and luteolin (0.0320 µg/mg). Among these, protocatechuic acid was the most abundant compound in the ethanolic extract of *M. autumnalis* and genistein in the *S. junceum*. See detailed LC‐MS/MS chromatograms in Figures 2 and 3.

**TABLE 1:** Quantitative LC‐MS/MS results of the ethanolic extracts of *S. junceum* and *M. autumnalis*.

Quantitative results (mg analyte/g extract)		
Analytes	*S. junceum*	*M. autumnalis*
Quinic acid	0.4673	<LOD
Fumaric acid	<LOD	<LOD
Aconitic acid	<LOD	<LOD
Gallic acid	<LOD	<LOD
Epigallocatechin	<LOD	<LOD
Protocatechuic acid	<LOD	0.6850
Catechin	<LOD	<LOD
Gentisic acid	<LOD	<LOD
Chlorogenic acid	<LOD	<LOD
Protocatechuic aldehyde	<LOD	<LOD
Tannic acid	<LOD	<LOD
Epigallocatechin gallate	<LOD	<LOD
Cynarin	<LOD	<LOD
4‐OH Benzoic acid	<LOD	<LOD
Epicatechin	<LOD	<LOD
Vanilic acid	<LOD	<LOD
Caffeic acid	0.1240	0.1080
Syringic acid	<LOD	<LOD
Vanillin	<LOD	<LOD
Syringic aldehyde	<LOD	<LOD
Daidzin	1.0920	<LOD
Epicatechin gallate	<LOD	<LOD
Piceid	<LOD	<LOD
*p*‐Coumaric acid	<LOD	<LOD
Ferulic acid‐D3‐ISh	N.A.	N.A.
Ferulic acid	<LOD	<LOD
Sinapic acid	<LOD	<LOD
Coumarin	<LOD	0.0280
Salicylic acid	0.0460	0.0570
Cynaroside	1.5240	<LOD
Miquelianin	<LOD	<LOD
Rutin‐D3‐IS	N.A.	N.A.
Rutin	<LOD	<LOD
isoquercitrin	<LOD	<LOD
Hesperidin	<LOD	<LOD
*o*‐Coumaric acid	<LOD	<LOD
Genistin	<LOD	<LOD
Rosmarinic acid	<LOD	<LOD
Ellagic acid	<LOD	<LOD
Cosmosiin	0.9550	<LOD
Quercitrin	<LOD	<LOD
Astragalin	<LOD	<LOD
Nicotiflorin	<LOD	<LOD
Fisetin	0.4830	<LOD
Daidzein	0.6980	<LOD
Quercetin‐D3‐IS	N.A.	N.A.
Quercetin	0.0220	<LOD
Naringenin	0.1660	0.0050
Hesperetin	0.0110	<LOD
Luteolin	0.8380	0.0320
Genistein	1.8280	<LOD
Kaempferol	0.0080	<LOD
Apigenin	0.2790	<LOD
Amentoflavone	<LOD	<LOD
Chrysin	<LOD	<LOD
Acacetin	0.2330	<LOD

LC‐MS/MS analysis demonstrated a pronounced qualitative and quantitative difference between the phenolic profiles of *S. junceum* and *M. autumnalis*. A total of 16 metabolites were identified in *S. junceum*, whereas only six compounds were detected in *M. autumnalis*, with four metabolites (caffeic acid, salicylic acid, naringenin, and luteolin) being common to both species. In terms of total quantified phenolic load, *S. junceum* exhibited nearly an order of magnitude higher accumulation than *M. autumnalis*, indicating a richer and more diverse phenolic and flavonoid composition. Detailed LC‐MS/MS chromatogram of *S. junceum* and *M. autumnalis* is shown in Figures 2 and 3, respectively.

The phytochemical profile of *S. junceum* was dominated by flavonoids, particularly isoflavones such as genistein, daidzin, and daidzein, together with flavones (luteolin, apigenin, acacetin) and flavonols (fisetin, quercetin, kaempferol). This structural diversity, combined with the relatively high individual concentrations of these compounds, suggests a strong capacity for radical scavenging, metal chelation, and enzyme inhibition, since flavonoids bearing multiple hydroxyl groups are known to interact efficiently with biological targets. In contrast, *M. autumnalis* was characterized mainly by phenolic acids, with protocatechuic acid as the major compound, accompanied by lower amounts of caffeic acid and only trace levels of flavonoids. This indicates that the antioxidant and enzyme inhibitory potential of this species may rely more on simple phenolic acids rather than on structurally complex flavonoids.

Interestingly, despite the higher total phenolic and flavonoid contents obtained for *M. autumnalis*, LC‐MS/MS revealed a lower number and total amount of individually quantified metabolites. Therefore, the higher TPC/TFC values of *M. autumnalis* should not be interpreted as a higher concentration of the targeted individual phenolics detected by LC‐MS/MS, but rather as a stronger total colorimetric response of the whole extract matrix. Conversely, the higher number and total amount of quantified compounds in *S. junceum* indicate a richer profile of the selected target metabolites included in the LC‐MS/MS panel. This apparent discrepancy may be attributed to the presence of polymeric phenolics, glycosylated derivatives, or other phenolic constituents that are not included in the targeted LC‐MS/MS library, as well as to the nonspecific nature of the Folin–Ciocalteu assay, which responds to all reducing substances [14, 26]. Therefore, the colorimetric assays reflect the overall reducing capacity of the extract, whereas LC‐MS/MS provides a more selective insight into the individual metabolite distribution. Moreover, the presence of common metabolites in both species indicates a shared core phenylpropanoid pathway, while the species‐specific compounds reflect metabolic specialization. The high abundance of genistein and related isoflavones in *S. junceum* is particularly noteworthy, as these compounds are well documented for their strong antioxidant, anti‐inflammatory, and enzyme inhibitory properties, which may significantly contribute to the biological activity of this extract [29, 30, 31]. Overall, the combined evaluation of spectrophotometric and LC‐MS/MS data clearly shows that TPC alone is not sufficient to describe the phytochemical richness of plant extracts. Instead, both the quantitative accumulation and the structural diversity of individual metabolites must be considered to better understand their biological potential.

### 3.2 Antioxidant Properties

Antioxidants protect biological systems against oxidative stress by scavenging free radicals, inhibiting their formation, and reducing oxidized intermediates through multiple mechanisms [14]. Therefore, the evaluation of antioxidant capacity requires the application of different analytical approaches reflecting these distinct modes of action. In the present study, the antioxidant activities of *S. junceum* and *M. autumnalis* were assessed using assays based on two complementary mechanisms. Radical scavenging ability was determined by DPPH radical scavenging assays, while the reducing power of the extracts was evaluated using the Fe^3+^ reducing assay, FRAP, and CUPRAC assays. Since each method measures a different aspect of antioxidant behavior, the combined use of these assays provides a more comprehensive and biologically relevant estimation of the overall antioxidant potential of the extracts [9].

In the DPPH radical scavenging assay, lower IC_50_ values indicate stronger radical scavenging activity. According to the results presented in Table 2, the antioxidant activity based on IC_50_ values was ranked from most active to least active as follows: α‐tocopherol (14.78 ± 5.50 µg/mL), BHT (39.63 ± 9.21 µg/mL), *M. autumnalis* ethanolic extract (60.16 ± 2.02 µg/mL), BHA (158.4 ± 2.83 µg/mL), and *S. junceum* ethanolic extract (813.50 ± 2.28 µg/mL). These results indicate that the ethanolic extract of *M. autumnalis* exhibits a higher radical scavenging capacity compared to the BHA. In contrast, although the ethanolic extract of *S. junceum* did not show a comparable level of activity, it was found to possess a low radical scavenging potential.

**TABLE 2:** Antioxidant activities of the ethanolic extracts of *M. autumnalis* and *S. junceum*.

	DPPH· scavenging assay (IC_50_)	CUPRAC assay (*λ*_450_)	FRAP assay (*λ*_595_)	Fe^3+^ reducing assay (*λ*_700_)
Ascorbic acid	—	2.301 ± 0.074^b^	2.041 ± 0.045^a^	—
BHA	158.4 ± 2.827	1.275 ± 0.090^d^	1.323 ± 0.048^b^	0.514 ± 0.052^a,b^
BHT	39.63 ± 9.212	2.641 ± 0.070^a^	2.065 ± 0.058^a^	0.404 ± 0.020^b^
α‐Tocopherol	14.78 ± 5.504	2.528 ± 0.072^a^	2.256 ± 0.106^a^	—
Trolox	—	2.339 ± 0.109^b^	2.089 ± 0.132^a^	0.591 ± 0.045^a^
*M. autumnalis*	60.16 ± 2.021	1.960 ± 0.0612^c^	2.098 ± 0.042^a^	0.597 ± 0.051^a^
*S. junceum*	813.5 ± 2.276	0.863 ± 0.051^e^	0.936 ± 0.164^c^	0.370 ± 0.065^b^

*Note:* DPPH radical scavenging results are expressed as IC_50_ (µg/mL), whereas CUPRAC, FRAP, and Fe^3+^ reducing power results are expressed as absorbance ± SD at 45 µg/mL. Values are presented as mean ± SD. Different superscript letters within the same column indicate significant differences (*p* < 0.05). “—” indicates not determined.

**FIGURE 4:**
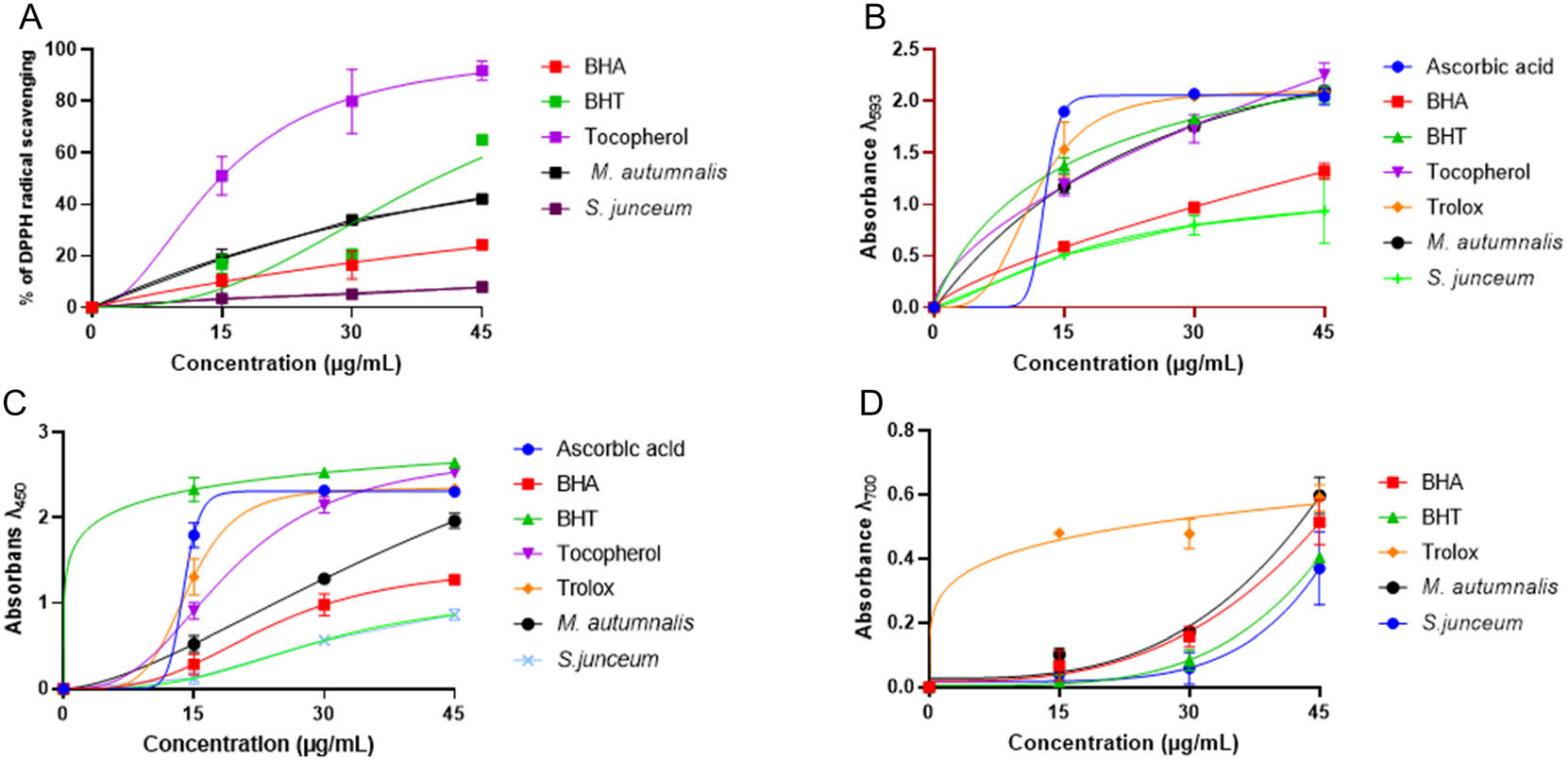
Graphical representation of the antioxidant analysis results of *S. junceum* and *M. autumnalis* extracts.

As a result of the CUPRAC, FRAP, and Fe^3+^ reducing assays, it was determined that the ethanolic extracts of *S. junceum* and *M. autumnalis* exhibited a pronounced reducing capacity at a concentration of 45 µg/mL and showed values comparable to those of standard antioxidants. According to the FRAP assay, the reducing capacity ranked from highest to lowest as α‐tocopherol, *M. autumnalis*, Trolox, BHT, ascorbic acid, BHA, and *S. junceum* (Figure 4). In the Fe^3+^ reducing assay, the *M. autumnalis* extract exhibited a reducing capacity very close to that of Trolox and comparable to the strongest reference antioxidant tested in this assay. In the CUPRAC assay, BHT and α‐tocopherol exhibited the highest reducing capacities and were statistically comparable (*p* > 0.05), followed by Trolox and ascorbic acid. Among the plant extracts, *M. autumnalis* showed significantly higher CUPRAC activity than *S. junceum* and BHA (*p* < 0.05), whereas *S. junceum* displayed the lowest reducing capacity. In the FRAP assay, *M. autumnalis* showed a reducing capacity statistically comparable to α‐tocopherol, Trolox, BHT, and ascorbic acid, whereas BHA and *S. junceum* formed lower statistical groups, with *S. junceum* exhibiting the weakest FRAP response. In the Fe^3+^ reducing assay, *M. autumnalis* showed a reducing capacity statistically comparable to Trolox, while both exhibited significantly higher activity than *S. junceum*. BHA showed an intermediate response, whereas BHT and *S. junceum* were grouped among the lower reducing activities.

Antioxidant assays are in good agreement with the LC‐MS/MS data. The antioxidant activity of *M. autumnalis* can be attributed to its phenolic acid‐rich profile, particularly protocatechuic and caffeic acids, which possess low molecular weight and multiple hydroxyl groups, facilitating efficient electron and hydrogen transfer. In contrast, although *S. junceum* contained a more diverse flavonoid and isoflavonoid profile, this did not translate into stronger DPPH radical scavenging activity. This finding suggests that antioxidant activity is not determined solely by the number or total amount of detected phenolic compounds, but also by their structural features, including hydroxylation pattern, glycosylation, conjugation, polarity, and reaction kinetics under the specific assay conditions. In particular, some isoflavones and flavonoid glycosides may exhibit lower hydrogen‐ or electron‐donating efficiency in the DPPH radical scavenging system compared with low‐molecular‐weight phenolic acids such as protocatechuic and caffeic acids [14]. Therefore, the weaker DPPH radical scavenging activity of *S. junceum* may be related to the qualitative nature and reactivity of its major metabolites rather than to phenolic abundance alone. Overall, these findings support the strong antioxidant potential of *M. autumnalis* and highlight the importance of the structural characteristics of individual metabolites in determining antioxidant behavior.

The antioxidant activity of the *M. autumnalis* root extract in the present study (DPPH radical scavenging IC_50_ = 60.16 µg/mL; CUPRAC = 1.96; FRAP = 2.098; Fe^3+^ reducing = 0.597) is in line with the findings of Uysal et al., who reported that the antioxidant capacity of this species varies markedly depending on plant part and extraction solvent, with the flower extracts exhibiting the highest activity and the leaf extracts the lowest [32]. Although their results were expressed as Trolox equivalents, the overall trend indicating a strong correlation between phenolic content and reducing power is consistent with our data. The comparatively high reducing capacity observed in our root extract, particularly its Fe^3+^ reducing activity, which was comparable to Trolox, suggests that this plant part also contains efficient electron‐donating phenolics. However, the stronger activities reported for the flower extracts by Uysal et al. support the view that antioxidant metabolites are unevenly distributed among plant organs [32]. These findings confirm that the antioxidant potential of *M. autumnalis* is organ‐dependent and largely governed by the qualitative phenolic profile rather than the total extract yield.

Compared with the study of Albahri et al., the antioxidant performance obtained in the present work was considerably higher in terms of radical scavenging efficiency and reducing power. In their report, the ethanolic leaf extract of *M. autumnalis* exhibited 54.94 ± 0.89% DPPH inhibition at 600 µg/mL, together with relatively low total phenolic and flavonoid contents (58.98 ± 7.40 mg GAE/g and 36.47 ± 0.87 mg QE/g, respectively) [4]. Despite this moderate phenolic level, their extract showed limited antioxidant effectiveness, whereas the stronger activity observed in the current study indicates that antioxidant capacity in *M. autumnalis* is more closely related to the qualitative phenolic profile and the distribution of highly active low‐molecular‐weight metabolites than to the total phenolic amount alone. This difference also supports the organ‐dependent accumulation of bioactive compounds and highlights the critical role of extraction strategy and metabolite composition in determining the overall antioxidant potential.

Compared with the study in which different flower and stem extracts of *S. junceum* were evaluated, their antioxidant capacity remained at moderate levels, as indicated by the DPPH' radical scavenging activity of the stem methanol extract (IC_50_ = 1.42 mg/mL), despite relatively high total phenolic (63.94 mg GAE/g) and flavonoid (61.33 mg QE/g) contents in the aqueous stem extract. They have reported that biological activity in *S. junceum* is not directly proportional to total phenolic quantity but is more strongly influenced by the qualitative composition of individual metabolites and the extraction strategy [33]. This observation is in agreement with the LC‐MS/MS profile, which revealed a metabolite pattern dominated by specific flavonoids and isoflavones known to interact effectively with cholinesterase enzymes.

In comparison with the literature data, the antioxidant activity of *S. junceum* obtained in the present study showed improved radical scavenging efficiency based on DPPH IC_50_ values. Previous reports indicated weak DPPH activity for *S. junceum* extracts, with IC_50_ values of 2327–7757 µg/mL, whereas our extract exhibited a substantially lower IC_50_ value. Since CUPRAC, FRAP, and Fe^3+^ reducing power results were expressed as absorbance values under assay‐specific conditions, direct quantitative comparison of these reducing assays with previous studies was avoided [34]. In contrast to the literature, where antioxidant activity was strongly associated with high total phenolic and flavonoid contents in aqueous extracts, the stronger activity observed here suggests that antioxidant performance is governed not only by total phenolic quantity but also by the qualitative composition of individual metabolites. This difference may be attributed to the extraction strategy and the LC‐MS/MS‐characterized flavonoid/isoflavonoid profile of the extract, indicating that specific bioactive constituents rather than bulk phenolic content play a decisive role in the antioxidant response.

### 3.3 Enzyme Inhibition Profile

AChE is a key enzyme in the central and peripheral nervous systems that rapidly hydrolyzes the neurotransmitter acetylcholine, thereby terminating nerve impulse transmission. Inactivation of AChE by various inhibitors leads to the accumulation of acetylcholine in the synaptic cleft and disruption of neurotransmission. Because acetylcholine levels are reduced in Alzheimer's disease, reversible AChE inhibitors are widely used in its treatment [35, 36, 37]. One such inhibitor, donepezil, which was used as the reference compound in this study, is a selective and reversible AChE inhibitor that increases acetylcholine levels, improves cognitive symptoms, and has the potential to slow disease progression [38, 39].

It should be noted that the IC_50_ values of crude plant extracts expressed in µg/mL cannot be directly compared with the molar potency of pure reference inhibitors such as donepezil and AZA. Crude extracts are complex mixtures containing multiple constituents with unknown active compound proportions, whereas the reference inhibitors are pure compounds. Therefore, the reference inhibitors were used as assay controls, and the extract IC_50_ values should be interpreted as preliminary screening results obtained under the applied experimental conditions rather than as evidence of superior potency over the standard drugs. According to the results, the cholinesterase inhibitory activities of the ethanolic extracts of *M. autumnalis* and *S. junceum* were evaluated using donepezil as the reference inhibitor (Table 3). In the AChE inhibition assay, the IC_50_ values were determined as 6.93 µg/mL for *M. autumnalis*, 12.22 µg/mL for donepezil, and 27.51 µg/mL for *S. junceum*. Among the tested extracts, *M. autumnalis* exhibited a more pronounced inhibitory effect against AChE, whereas *S. junceum* showed weaker AChE inhibition, as indicated by its higher IC_50_ value. The AChE inhibitory activity of *M. autumnalis* in the present study was markedly stronger than that reported previously for the same species, where IC_50_ values of 5.1–48.1 mg/mL were obtained depending on the plant part and assay conditions [40].

**TABLE 3:** Enzyme inhibition activities of the ethanolic extracts of *M.* *autumnalis* and *S. junceum*.

Enzyme samples	IC_50_, μg/mL							
	hCA I	*r* ^2^	hCA II	*r* ^2^	AChE	*r* ^2^	BChE	*r* ^2^
*M. autumnalis*	23.63	0.9325	19.34	0.9840	6.93	0.9890	64.70	0.8320
*S. junceum*	180.60	0.9702	54.80	0.9653	27.51	0.9916	13.32	0.9690
References	55.10	0.9963	49.80	0.9840	12.22	0.9996	8.82	0.9836

*Note:* Donepezil was used as the reference inhibitor for AChE and BChE, whereas acetazolamide was used as the reference inhibitor for hCA I and hCA II.

Different inhibition profiles were observed in the BChE assay. The IC_50_ values were 64.70 µg/mL for *M. autumnalis*, 8.82 µg/mL for donepezil, and 13.32 µg/mL for *S. junceum* (Table 3). These results indicate that *M. autumnalis* did not show the same level of inhibitory activity against BChE as it did against AChE. In contrast, *S. junceum* displayed stronger BChE inhibition than *M. autumnalis* under the applied experimental conditions, suggesting a relatively more selective BChE inhibitory profile among the tested extracts. However, since the plant extracts are complex crude matrices and donepezil is a pure reference inhibitor, direct therapeutic potency comparisons should be avoided.

This difference is consistent with the LC‐MS/MS profiles of the extracts. The phenolic acid‐ and coumarin‐dominated composition of *M. autumnalis*, characterized by low‐molecular‐weight compounds such as protocatechuic and caffeic acids, may facilitate access to the narrow active gorge of AChE, resulting in stronger inhibition [41]. Conversely, the flavonoid‐ and isoflavone‐rich profile of *S. junceum* (genistein, daidzein, luteolin, apigenin) with bulkier molecular structures may favor interaction with the wider active site of BChE, leading to its higher inhibition activity toward this enzyme [42]. The cholinesterase inhibitory activity of *S. junceum* in the present study was markedly stronger than previously reported values [34]. Earlier studies indicated IC_50_ values of 46.69 mg/mL for AChE and 76.39 mg/mL for BChE for the most active extracts [34]. Compared with previously reported cholinesterase inhibitory data for *S. junceum* extracts, the present extract exhibited lower IC_50_ values under our experimental conditions. However, extraction solvent, plant part, assay conditions, and enzyme source may differ between studies, these comparisons should be interpreted cautiously.

Overall, *M. autumnalis* showed a more pronounced AChE inhibition profile, whereas *S. junceum* exhibited relatively stronger inhibition against BChE among the tested extracts. These findings suggest that the two extracts may differ in their cholinesterase inhibition patterns and could serve as preliminary sources for further bioactivity‐guided investigations. However, considering their known toxic properties and the crude nature of the tested extracts, further studies are required to identify the active constituents, determine their selectivity, and evaluate their safety before any therapeutic relevance can be proposed.

Carbonic anhydrase is an important metalloenzyme that efficiently catalyzes the conversion of CO_2_ into bicarbonate (HCO_3_^−^) in the metabolism [43]. Through the action of carbonic anhydrase, CO_2_ is rapidly and tightly converted into bicarbonate, thereby preventing potentially harmful effects. While bicarbonate has limited solubility in water, CO_2_ can dissolve very rapidly and enter the bloodstream, which may lead to various health problems [43, 44]. The enzyme plays a critical role in maintaining the CO_2_‐bicarbonate equilibrium in aqueous environments and acts as a buffer against pH fluctuations [45]. Inhibition of carbonic anhydrase has therapeutic applications in the treatment of several diseases, including infections, glaucoma, and cancer [7]. AZA, which is used in the treatment of neurological disorders such as glaucoma, hypertension, and epileptic seizures, is a clinically approved carbonic anhydrase inhibitor [46]. In this study, AZA was used as the reference compound. Evaluation of the plant extracts revealed the following inhibitory activity ranking for the hCA I enzyme (Table 3) *M. autumnalis* extract (IC_50_ = 23.63 µg/mL) > AZA (IC_50_ = 55.10 µg/mL) > *S. junceum* extract (IC_50_ = 180.6 µg/mL). For the hCA II enzyme, the inhibitory activity ranking was determined as *M. autumnalis* extract (IC_50_ = 19.34 µg/mL) > AZA (IC_50_ = 49.80 µg/mL) > *S. junceum* extract (IC_50_ = 54.80 µg/mL). These results suggest that *M. autumnalis* had a more pronounced inhibitory effect on both hCA isoenzymes than *S. junceum* under the applied assay conditions. However, since AZA is a pure reference inhibitor and the plant extracts are complex mixtures, direct potency comparisons should be avoided. Moreover, its inhibitory effects on key enzymes—particularly hCA II, which is associated with diabetes, glaucoma, and Alzheimer's disease—are highly noteworthy [47].

Carbonic anhydrase inhibition by plant extracts is strongly influenced by the presence of phenolic acids, flavonoids, and other secondary metabolites capable of interacting with the active site of the enzyme or coordinating with the catalytic Zn^2+^ ion [48, 49]. The LC‐MS/MS analysis performed in the present study revealed clear differences in the phytochemical profiles of *M. autumnalis* and *S. junceum*, which may explain their distinct inhibitory activities against hCA I and hCA II. The superior inhibitory effect of *M. autumnalis* can be attributed to the presence of phenolic acids such as protocatechuic acid and caffeic acid, together with coumarin derivatives. These compounds are well known for their ability to inhibit carbonic anhydrase through different mechanisms [48, 50, 51].

In contrast, although *S. junceum* contained a higher number and total amount of flavonoids, its inhibitory activity against both isoenzymes was lower. This may be explained by the structural characteristics of the major compounds identified in this extract, particularly isoflavones such as genistein, daidzin, and daidzein, as well as flavones including luteolin and apigenin. These molecules possess bulky and highly substituted structures that may limit their penetration into the narrow active site channel of carbonic anhydrase. Instead of directly coordinating to the Zn^2+^ ion, flavonoids generally bind at the entrance of the active site through hydrogen bonding and hydrophobic interactions, resulting in weaker inhibition compared with smaller phenolic acids or coumarin derivatives [51, 52, 53, 54]. Moreover, the stronger inhibition of hCA II compared to hCA I by *M. autumnalis* is noteworthy, since hCA II has a wider and more hydrophilic active site, which favors the binding of small polar phenolic compounds [54]. This observation further supports the role of the identified phenolic acids as the main contributors to the activity.

Overall, the CA inhibition results support the phytochemical profile obtained by LC‐MS/MS and identify *M. autumnalis* as a promising source of natural carbonic anhydrase inhibitors. To the best of our knowledge, this is the first study that determines the human carbonic anhydrase inhibition ability of *M. autumnalis*. In addition, *S. junceum* has previously been investigated for several enzyme inhibitory effects, including cholinesterase and α‐glucosidase inhibition, no data are available on its effects on carbonic anhydrase isoenzymes.

### 3.4 Antimicrobial Properties

The antimicrobial activities of the ethanolic extracts of *M. autumnalis* and *S. junceum* were evaluated using the disc diffusion method. Sterile paper disks (6 mm in diameter) were impregnated with different concentrations of the extracts and placed on agar plates previously inoculated with Gram‐positive and Gram‐negative bacterial strains, including *S. aureus*, *E. coli*, *P. aeruginosa*, and *K. pneumoniae*. According to the results, the extract of *M. autumnalis* exhibited preliminary antibacterial activity under the applied conditions against *E. coli* and *S. aureus*, producing detectable inhibition zones of 11 mm at 80 µg/disc and 11 mm at 40 µg/disc, respectively. No inhibition was observed against *P. aeruginosa* or *K. pneumoniae*. In contrast, the ethanolic extract of *S. junceum* inhibited the growth of *E. coli* and *K. pneumoniae*, with inhibition zone diameters of 13 and 11 mm at concentrations of 80 and 40 µg/disc, respectively. From positive controls, tetracycline at 20 µg/disc, 21, 18, 15 and 8 mm zones were recorded for *S. aureus*, *E. coli*, *K. pneumoniae* and *P. aeruginosa*, respectively. Any growth inhibition was recorded with DMSO (3%).

The difference in antibacterial spectra between the two extracts may be attributed to their distinct phytochemical compositions and possible mechanisms of action. Although Gram‐negative bacteria are generally more resistant due to the presence of an outer membrane, the activity of *M. autumnalis* against both *S. aureus* and *E. coli* suggests that its antibacterial effect is not limited to cell wall disruption and may involve more general mechanisms such as membrane permeability alteration, intracellular target interaction, or redox imbalance [55].

In contrast, the ethanolic extract of *S. junceum* exhibited activity against *K. pneumoniae*, a highly encapsulated Gram‐negative bacterium [56, 57]. Considering the flavonoid‐ and isoflavone‐rich profile of *S. junceum*, these compounds may enhance cell envelope permeability or interfere with the integrity of the capsule, thereby increasing bacterial susceptibility, as reported in the literature [58]. A study represents a flavonoid‐rich fruit fraction of *M. autumnalis*, which exhibited an extremely strong activity with a MIC value of 0.135 µg/mL [59]. Plant‐derived phenolics and flavonoids have been reported to exert antibacterial effects through multiple mechanisms, including disruption of bacterial cell membranes, alteration of membrane permeability, inhibition of microbial enzymes, interference with quorum sensing and biofilm formation, and interactions with bacterial surface‐associated structures such as extracellular or capsular polysaccharides [29, 30, 31, 60, 61]. However, these mechanisms were not directly investigated in the present study [62]. Therefore, the antibacterial activity observed for *S. junceum* should be interpreted only as a preliminary screening result, and further mechanistic studies are required to clarify the exact mode of action [32, 33, 34, 63, 64].

## 4 Conclusions

The present study demonstrated that *M. autumnalis* and *S. junceum* possess distinct phytochemical profiles associated with different biological activities. The combined evaluation of spectrophotometric analyses, targeted LC‐MS/MS profiling, and biological assays indicated that qualitative metabolite composition plays a more important role in determining bioactivity than TPC alone. Although both extracts exhibited promising antioxidant, enzyme inhibitory, and preliminary antibacterial activities, their biological responses differed according to their characteristic metabolite profiles. These findings provide a comparative phytochemical and biological basis for the further investigation of these species as potential sources of bioactive natural products. However, because the present results are based on in vitro assays using crude extracts, further studies involving bioactivity‐guided fractionation, isolation and characterization of active constituents, mechanistic investigations, standardized antimicrobial evaluation, toxicity assessment, and in vivo validation are required before any therapeutic applications can be considered.

## Funding

This study was supported by the Türkiye Bilimsel ve Teknolojik Araştırma Kurumu.

## Conflicts of Interest

The authors declare no conflicts of interest.

## Supporting information

Supplementary Material

## Data Availability

The data that support the findings of this study are available from the corresponding author upon reasonable request.
