# Supplementary material for: Metabolite‐Driven Biological Activity of Mandragora autumnalis and Spartium junceum: A Comparative LC‐MS/MS Study on Antioxidant, Cholinesterase, and Carbonic Anhydrase Inhibition
Source: ChemistryOpen. 2026 Aug 13;15(8):e70269. doi: 10.1002/open.70269 (PMC13470032; doi:10.1002/open.70269)
Supplement: Supplementary file 1 [file OPEN-15-e70269-s001.pdf]

Supporting Information

Metabolite-Driven Biological Activity of *Mandragora autumnalis* and *Spartium junceum*: A Comparative LC–MS/MS Study on Antioxidant, Cholinesterase, and Carbonic Anhydrase Inhibition

Kübra ASLAN[1], Esra ELBIR[2], Yusuf BICAKCI[1], Sılanur OZDOGAN[2], Mustafa Abdullah YILMAZ[3], Ilhami GULCIN[1,4]

[1]*Atatürk University, Department of Chemistry, Faculty of Science, Erzurum, Türkiye*

[2] *Atatürk University, Department of Molecular Biology and Genetics, Faculty of Science, Erzurum, Türkiye*

[3]*Dicle Unıversity, Department of Analytical Chemistry, Faculty of Pharmacy, Diyarbakir, Türkiye*

[4]*Agri Ibrahim Cecen University, Rectorate, Agri, Türkiye*

Corresponding address:

Atatürk University

Department of Chemistry

Faculty of Science

Erzurum- Türkiye

kubra.aslan@atauni.edu.tr, igulcin@atauni.edu.tr

Table of Contents	Page
**Materials**	4
**Table S1.** Validation Summart Report	5
**Table S1.** Validation Summart Report (Continued)	6
**Table S1.** Validation Summart Report (Continued)	7
**Table S1.** Validation Summart Report (Continued)	8
**Table S1.** Validation Summart Report (Continued)	9
**Table S1.** Validation Summart Report (Continued)	10
**References**	11

Materials

Reference phytochemical standards (quinic acid, gallic acid, fumaric acid, aconitic acid, protocatechuic acid, epigallocatechin, gentisic acid, catechin, chlorogenic acid, tannic acid, protocatechuic aldehyde, epigallocatechin gallate, epicatechin, 4-OH-benzoic acid, vanillic acid, syringic acid, caffeic acid, vanillin, daidzin, syringaldehyde, epicatechin gallate, ferulic acid, sinapic acid, p-coumaric acid, coumarin, cynaroside, salicylic acid, rutin, miquelianin, isoquercitrin, o-coumaric acid, hesperidin, genistin, ellagic acid, rosmarinic acid, cosmosiin, astragalin, quercitrin, nicotiflorin, daidzein, fisetin, quercetin, luteolin, naringenin, hesperetin, apigenin, genistein, kaempferol, chrysin, amentoflavone, and acacetin) were purchased from Sigma Aldrich (Steinheim, Germany). 1,5-Dicaffeoylquinic acid and the internal standards of rutin-D3, ferulic acid-D3, and quercetin-D3 were obtained from TRC (Toronto, Ontario, Canada).

Table S1. Validation Summart Report

No	Analyte	RT	Procursor ion	Product Ion	Ionization Mode	Calibration Equation	R^2	Interday RSD	Intraday RSD	Linaerity Range	LOD/LOQ	Intraday Recovery	Interday Recovery
1	Quinic acid	3.0	190.8	93.0	Neg	y=−0.0129989 + 2.97989×	0.996	0.69	0.51	0.1-5	25.7/33.3	1.0011	1.0083
2	Fumaric aid	3.9	115.2	40.9	Neg	y=−0.0817862 + 1.03467×	0.995	1.05	1.02	1-50	135.7/167.9	0.9963	1.0016
3	Aconitic acid	4.0	172.8	129.0	Neg	y=−0.7014530 + 32.9994×	0.971	2.07	0.93	0.1-5	16.4/31.4	0.9968	1.0068
4	Gallic acid	4.4	168.8	79.0	Neg	y = 0.0547697 + 20.8152×	0.999	1.60	0.81	0.1-5	13.2/17.0	1.0010	0.9947
5	Epigallocatechin	6.7	304.8	219.0	Neg	y=−0.00494986 + 0.0483704×	0.998	1.22	0.73	1-50	237.5/265.9	0.9969	1.0040
6	Protocatechuic acid	6.8	152.8	108.0	Neg	y = 0.211373 + 12.8622×	0.957	1.43	0.76	0.1-5	21.9/38.6	0.9972	1.0055
7	Catechin	7.4	288.8	203.1	Neg	y=−0.00370053 + 0.431369×	0.999	2.14	1.08	0.2-10	55.0/78.0	1.0024	1.0045
8	Gentisic acid	8.3	152.8	109.0	Neg	y=−0.0238983 + 12.1494×	0.997	1.81	1.22	0.1-5	18.5/28.2	0.9963	1.0077
9	Chlorogenic acid	8.4	353.0	85.0	Neg	y = 0.289983 + 36.3926×	0.995	2.15	1.52	0.1-5	13.1/17.6	1.0000	1.0023

No	Analyte	RT	Procursor ion	Product Ion	Ionization Mode	Calibration Equation	R^2	Interday RSD	Intraday RSD	Linaerity Range	LOD/LOQ	Intraday Recovery	Interday Recovery
10	Protocatechuic aldehyde	8.5	137.2	92.0	Neg	y = 0.257085 + 25.4657×	0.996	2.08	0.57	0.1-5	15.4/22.2	1.0002	0.9988
11	Tannic acid	9.2	182.8	78.0	Neg	y = 0.0126307 + 26.9263×	0.999	2.40	1.16	0.05-2.5	15.3/22.7	0.9970	0.9950
12	Epigallocatechin gallate	9.4	457.0	305.1	Neg	y=−0.0380744 + 1.61233×	0.999	1.30	0.63	0.2-10	61.0/86.0	0.9981	1.0079
13	1,5-dicaffeoylquinic acid	9.8	515.0	191.0	Neg	y=−0.0164044 + 16.6535×	0.999	2.42	1.48	0.1-5	5.8/9.4	0.9983	0.9997
14	4-OH Benzoic acid	10.5	137,2	65.0	Neg	y=−0.0240747 + 5.06492×	0.999	1.24	0.97	0.2-10	68.4/88.1	1.0032	1.0068
15	Epicatechin	11.6	289.0	203.0	Neg	y=−0.0172078 + 0.0833424×	0.996	1.47	0.62	1-50	139.6/161.6	1.0013	1.0012
16	Vanilic acid	11.8	166.8	108.0	Neg	y=−0.0480183 + 0.779564×	0.999	1.92	0.76	1-50	141.9/164.9	1.0022	0.9998
17	Caffeic acid	12.1	179.0	134.0	Neg	y = 0.120319 + 95.4610×	0.999	1.11	1.25	0.05-2.5	7.7/9.5	1.0015	1.0042
18	Syringic acid	12.6	196.8	166.9	Neg	y=−0.0458599 + 0.663948×	0.998	1.18	1.09	1-50	82.3/104.5	1.0006	1.0072
19	Vanillin	13.9	153.1	125.0	Poz	y = 0.00185898 + 20.7382×	0.996	1.10	0.85	0.1-5	24.5/30.4	1.0009	0.9967

No	Analyte	RT	Procursor ion	Product Ion	Ionization Mode	Calibration Equation	R2	Interday RSD	Intraday RSD	Linaerity Range	LOD/LOQ	Intraday Recovery	Interday Recovery
20	Syringic aldehyde	14.6	181.0	151.1	Neg	y=−0.0128684 + 7.90153×	0.999	2.51	0.77	0.4-20	19.7/28.0	1.0001	0.9964
21	Daidzin	15.2	417.1	199.0	Poz	y = 9.45747 + 152.338×	0.996	2.25	1.32	0.05-2.5	7.0/9.5	0.9955	1.0017
22	Epicatechin gallate	15.5	441.0	289.0	Neg	y=−0.0142216 + 1.06768×	0.997	1.63	1.28	0.1-5	19.5/28.5	0.9984	0.9946
23	Piceid	17.2	391.0	135/106.9	Poz	y = 0.00772525 + 25.4181×	0.999	1.94	1.16	0.05-2.5	13.8/17.8	1.0042	0.9979
24	p-Coumaric acid	17.8	163.0	93.0	Neg	y = 0.0249034 + 18.5180×	0.999	1.92	1.43	0.1-5	25.9/34.9	1.0049	1.0001
25	Ferulic acid-D3 ISh	18.8	196.2	152.1	Neg	N.A.	N.A.	N.A.	N.A.	N.A.	N.A.	N.A.	N.A.
26	Ferulic acid	18.8	192.8	149.0	Neg	y=−0.0735254 + 1.34476×	0.999	1.44	0.53	1-50	11.8/15.6	0.9951	0.9976
27	Sinapic acid	18.9	222.8	193.0	Neg	y=−0.0929932 + 0.836324×	0.999	1.45	0.52	0.2-10	65.2/82.3	1.0031	1.0037
28	Coumarin	20.9	146.9	103.1	Poz	y = 0.0633397 + 136.508×	0.999	2.11	1.54	0.05-2.5	214.2/247.3	0.9950	0.9958

No	Analyte	RT	Procursor ion	Product Ion	Ionization Mode	Calibration Equation	R2	Interday RSD	Intraday RSD	Linaerity Range	LOD/LOQ	Intraday Recovery	Interday Recovery
29	Salicylic acid	21.8	137.2	65.0	Neg	y = 0.239287 + 153.659×	0.999	1.48	1.18	0.05-2.5	6.0/8.3	0.9950	0.9998
30	Cynaroside	23.7	447.0	284.0	Neg	y = 0.280246 + 6.13360×	0.997	1.56	1.12	0.05-2.5	12.1/16.0	1.0072	1.0002
31	Miquelianin	24.1	477.0	150.9	Neg	y=-0.00991585 + 5.50334×	0.999	1.31	0.95	0.1-5	10.6/14.7	0.9934	0.9965
32	Rutin-D3-ISh	25.5	612.2	304.1	Neg	N.A.	N.A.	N.A.	N.A.	N.A.	N.A.	N.A.	N.A.
33	Rutin	25.6	608.9	301.0	Neg	y=-0.0771907 + 2.89868×	0.999	1.38	1.09	0.1-5	15.7/22.7	0.9977	1.0033
34	isoquercitrin	25.6	463.0	271.0	Neg	y=-0.111120 + 4.10546×	0.998	2.13	0.78	0.1-5	8.7/13.5	1.0057	0.9963
35	Hesperidin	25.8	611.2	449.0	Poz	y = 0.139055 + 13.2785×	0.999	1.84	1.35	0.1-5	19.0/26.0	0.9967	1.0043
36	o-Coumaric acid	26.1	162.8	93.0	Neg	y = 0.00837193 + 11.2147×	0.999	2.11	1.46	0.1-5	31.8/40.4	1.0044	0.9986
37	Genistin	26.3	431.0	239.0	Neg	y = 1.65808 + 7.57459×	0.991	2.01	1.28	0.1-5	14.9/21.7	1.0062	1.0047
38	Rosmarinic acid	26.6	359.0	197.0	Neg	y=-0.0117238 + 8.04377×	0.999	1.24	0.86	0.1-5	16.2/21.2	1.0056	1.0002

No	Analyte	RT	Procursor ion	Product Ion	Ionization Mode	Calibration Equation	R2	Interday RSD	Intraday RSD	Linaerity Range	LOD/LOQ	Intraday Recovery	Interday Recovery
39	Ellagic acid	27.6	301.0	284.0	Neg	y = 0.00877034 + 0.663741×	0.999	1.57	1.23	0.4-20	56.9/71.0	1.0005	1.0048
40	Cosmosiin	28.2	431.0	269.0	Neg	y=-0.708662 + 8.62498×	0.998	1.65	1.30	0.1-5	6.3/9.2	0.9940	0.9973
41	Quercitrin	29.8	447.0	301.0	Neg	y=-0.00153274 + 3.20368×	0.999	2.24	1.16	0.1-5	4.8/6.4	0.9960	0.9978
42	Astragalin	30.4	447.0	255.0	Neg	y = 0.00825333 + 3.51189×	0.999	2.08	1.72	0.1-5	6.6/8.2	0.9968	0.9957
43	Nicotiflorin	30.6	592.9	255.0/284.0	Neg	y = 0.00499333 + 2.62351×	0.999	1.48	1.23	0.05-2.5	11.9/16.7	0.9954	1.0044
44	Fisetin	30.6	285.0	163.0	Neg	y = 0.0365705 + 8.09472×	0.999	1.75	1.19	0.1-5	10.1/12.7	0.9980	1.0042
45	Daidzein	34.0	253.0	223.0	Neg	y=-0.0329252 + 6.23004×	0.999	2.18	1.73	0.1-5	9.8/11.6	0.9926	0.9963
46	Quercetin-D3-ISh	35.6	304.0	275.9	Neg	N.A.	N.A.	N.A.	N.A.	N.A.	N.A.	N.A.	N.A.
47	Quercetin	35.7	301.0	272.9	Neg	y=+0.00597342 + 3.39417×	0.999	1.89	1.38	0.1-5	15.5/19.0	0.9967	0.9971
48	Naringenin	35.9	270.9	119.0	Neg	y=-0.00393403 + 14.6424×	0.999	2.34	1.69	0.1-5	2.6/3.9	1.0062	1.0020

No	Analyte	RT	Procursor ion	Product Ion	Ionization Mode	Calibration Equation	R2	Interday RSD	Intraday RSD	Linaerity Range	LOD/LOQ	Intraday Recovery	Interday Recovery
49	Hesperetin	36.7	301.0	136.0/286.0	Neg	y=+0.0442350 + 6.07160×	0.999	2.47	2.13	0.1-5	7.1/9.1	0.9998	0.9963
50	Luteolin	36.7	284.8	151.0/175.0	Neg	y=-0.0541723 + 30.7422×	0.999	1.67	1.28	0.05-2.5	2.6/4.1	0.9952	1.0029
51	Genistein	36.9	269.0	135.0	Neg	y=-0.00507501 + 12.1933×	0.999	1.48	1.19	0.05-2.5	3.7/5.3	1.0069	1.0012
52	Kaempferol	37.9	285.0	239.0	Neg	y=-0.00459557 + 3.13754×	0.999	1.49	1.26	0.05-2.5	10.2/15.4	0.9992	0.9990
53	Apigenin	38.2	268.8	151.0/149.0	Neg	y = 0.119018 + 34.8730×	0.998	1.17	0.96	0.05-2.5	1.3/2.0	0.9985	1.0003
54	Amentoflavone	39.7	537.0	417.0	Neg	y = 0.727280 + 33.3658×	0.992	1.35	1.12	0.05-2.5	2.8/5.1	0.9991	1.0044
55	Chrysin	40.5	252.8	145.0/119.0	Neg	y=-0.0777300 + 18.8873×	0.999	1.46	1.21	0.05-2.5	1.5/2.8	0.9922	1.0050
56	Acacetin	40.7	283.0	239.0	Neg	y=-0.559818 +	0.997	1.67	1.28	0.02-1	1.5/2.5	0.9949	1.0011

						163.062×							

References

[1] M.A. Yılmaz (2020). Simultaneous quantitative screening of 53 phytochemicals in 33 species of medicinal and aromatic plants: A detailed, robust and comprehensive LC–MS/MS method validation. *Ind. Crops Prod.* **149**, 112347.
